# Public Comfort Stations

**Published:** 1912-02

**Authors:** 


### Public Comfort Stations.

THE BUFFALO MEDICAL JOURNAL advocates the establishment of a number of toilet and rest rooms at convenient points, in all of the cities in its territory. The JOURNAL asks the coöperation of the newspapers in developing public sentiment and local legislation along this line. The reform is too clearly within the domain of health, decency and philanthropy, to require argument. Such stations should be absolutely free; they should be properly attended to insure cleanliness; they should be open all the time; they should be not only water closets but lavatories and places where the tired and sick may rest and receive emergency care; we can see no objection to including provision for persons wishing to eat luncheon which they carry, a supply of pure water, etc.

Whether soft drinks, light refreshments and confections should be sold at such places or not, is a matter on which we have no special opinion. This reminds us, however: Delaware Park, Buffalo, is a very pleasant spot especially at the close of a summer day and, when one gets hungry it is very convenient to go to the Casino for supper. But, somehow, after doing so a couple of times, there is tendency to go home or down town for supper. On Sunday afternoons, it is a pleasure to see crowds of people in the Park and to feel that it is the recreation place for the poor even more than for the rich. It does one's heart good to see working men with their wives and a long following of children. Did you ever watch such a family party file onto the verandah of the Casino, in anticipation of an ice cream soda and note the man's consternation when he was charged ten cents instead of the customary five? If there is anything in the theory that the parks are for the masses, is it not worth the while of the Commissioners to see that good quality and popular prices prevail? Or if the ten cent charge is to persist, why not bring the quality above the three cent mark?

